# Development of a Group Judgment Process for Forecasts of Health Care Innovations

**DOI:** 10.1001/jamanetworkopen.2018.5108

**Published:** 2018-11-21

**Authors:** Paul G. Shekelle, Dana P. Goldman

**Affiliations:** 1 Southern California Evidence-Based Practice Center Site at the RAND Corporation, Santa Monica, California; 2 Greater Los Angeles Veterans Affairs Healthcare System, Los Angeles, California; 3 Public Policy, Pharmacy, and Economics, University of Southern California, Los Angeles

## Abstract

**Importance:**

Health care costs have increased substantially over the past few decades, in part owing to the development and diffusion of new medical treatments. Forecasting potential future technologic innovations can allow for more informed planning.

**Objective:**

To assess the predictive validity of a structured formal method for forecasting future technologic innovations in health care.

**Design, Setting, and Participants:**

This pilot study combined an untested, unvalidated combination of a consensus process and group judgment process to evaluate forecasts made in 2001 for technologic innovations by 2021 in Alzheimer disease (AD) and cardiovascular disease (CVD). Six experts in AD and 7 experts in CVD composed the judgment group. The study was conducted in 2017-2018.

**Main Outcomes and Measures:**

Year 2001 forecasts for 2021 that were judged by experts as being close to correct, directionally correct, or not correct, as well as innovations that occurred since 2001 that were not predicted.

**Results:**

Four forecasts of innovations in AD, each considered to be between 30% and 40% likely to be achieved by 2021, were judged to be close to correct. One forecast was considered to be directionally correct, with a likelihood of occurrence of 40%, in that it was overoptimistic. One innovation that occurred was missed: new imaging techniques (amyloid β plaque and tau tangle positron emission tomographic imaging). Five forecasts of CVD innovations were considered to be at least 50% likely to occur by 2021, and of these, 2 were judged to be close to correct, 1 was judged as being directionally correct, and 2 were judged as being not correct (although in one of these forecasts, the overarching innovation has been achieved but with a different noninvasive imaging modality). Of 7 additional forecasts considered to be less likely to be achieved by 2021, 4 were judged to be close to correct and 3 were judged as being directionally correct. Two innovations occurred but were missed: transcatheter aortic valve replacement and cardiac resynchronization therapy. Across both conditions, 15 of 17 innovations forecasted were judged to be close to correct or directionally correct, 2 were judged to be incorrect, and there were 3 missed innovations.

**Conclusions and Relevance:**

Expert elicitation provided a useful, but not fully accurate, lens into future innovation.

## Key Points

**Question:**

What is the accuracy of future predictions of technologic breakthrough innovations in the next 20 years?

**Findings:**

In 2001, a diverse group of research and clinical experts was assembled to predict the likelihood of various breakthrough innovations in the next 20 years in Alzheimer disease and cardiovascular disease. In 2017, another group of clinical experts in each condition was assembled to judge the accuracy of these predictions and found that 15 of 17 predictions were judged to have been close to correct or directionally correct, 2 predictions were judged to be incorrect, and 3 breakthrough innovations occurred that were not predicted.

**Meaning:**

This method produced predictions that were good but not completely accurate; further work is needed to improve methods of innovation forecasting.

## Teaser

This pilot study assesses the accuracy of predictions made in 2001 by expert clinicians and researchers on technologic innovations in therapy for Alzheimer disease and cardiovascular disease by 2021.

## Introduction

“Predicting the future is a fool’s errand” is a popular saying. But not anticipating the future, at least with respect to health care, is also foolish. Health care is one of the largest expenses in nearly every country; in the United States it accounts for nearly 1 of every 5 dollars spent and 1 in every 4 dollars spent by the federal government. How those costs may change over time is important to both governmental policymakers and individuals.

Furthermore, much of this growth in health care spending is driven by new technology,^1^ which shows up most readily in the form of increased prices and intensity of service use.^2^ Being able to anticipate innovations that may be costly or influence demographic trends in morbidity and mortality allows us to plan for their diffusion and reimbursement and to design incentives to encourage value rather than just more spending.^3,4^ Thus, predicting future innovations need not be perfect to be useful: it only needs to encourage useful policy changes.

Having accepted that some attempt at forecasting innovations should be done, the question becomes how. Nearly everything identified by standard computerized database searches using title words such as *predict*, *future*, *innovations*, or *breakthroughs* yields commentaries written by 1 or 2 authors and consists of the authors’ speculation about what may happen, usually without reference to probability or time frame. One exception is “The Future of Healthcare,” published in 2015 by *The Economist*,^5^ which states that they “empaneled experts in the field of healthcare to predict what technologies and innovations we will see in the near term (the next 5 years) and in the long term (25 years and beyond)”^5^ and then “surveyed global business leaders in and around the healthcare sector on whether they agree with the panel’s views.”^5^ Results are presented in terms of proportions of respondents who indicate that the innovation will be a widespread reality in the designated period. Findings for innovations in the next 5 years included portable medical devices for professionals (38.9% of respondents), health sensors for consumers (37.7%), the internet of things in health care (34.7%), and 3-dimensional printing (32.4%). Findings for innovations in the next 25 years included telemedicine (43.9% of respondents agreeing that this will be a widespread reality), hospital redesign (39.6%), precision medicine (34.2%), and devices inside the body (nanotechnology) (28.4%). However, no assessment of the validity of these latter predictions will be possible until near 2040. We have been unable to identify studies evaluating any methods of forecasting future innovations.

We report herein a unique pilot study, in that 17 years ago, we used a formal method with nationally recognized biomedical experts to forecast future health care breakthrough innovations that might occur in the next 20 years. We recently assessed those predictions for accuracy and make suggestions for further work to improve the methods of innovation forecasting.

## Methods

Almost 20 years ago in response to a request from the Centers for Medicare & Medicaid Services, we developed a quantitative method that combined lessons learned from evidence-based medicine literature searching techniques regarding horizon scanning, with focused multidisciplinary expert judgment. The method consists of selecting a diverse group of experts in the condition, generating a list of potential innovations, performing a literature review on these innovations, and then conducting a 1-day face-to-face panel meeting during which each potential innovation is discussed and then formally rated for its likelihood of occurrence in the next 20 years.^6^

In selecting the experts, we sought individuals with a broad range of expertise, including clinicians and basic scientists. Experts were selected based on their having been authors of relevant publications, the advice of local subject matter experts, and input from the sponsor, the Centers for Medicare & Medicaid Services. The face-to-face meeting combines features of the nominal group process to list and define potential innovations for further discussion, an informal group process to discuss the evidence and opinion regarding each topic, and formal voting to develop specific estimates for the likelihood of the innovation’s occurring in a predetermined time frame—in this case, 20 years. Each innovation was discussed in terms of the target population to whom the innovation would apply; the likelihood of the innovation occurring, meaning being in widespread clinical use in the next 20 years (eg, 10% likely, 20% likely); the expected effect on morbidity and mortality of the innovation; and an estimate of the cost (in current dollars) of the innovation. We used the median as the measure of the group estimate of likelihood.^7^ This process was modified from an established group judgment process used to assess the clinical effectiveness of interventions.^8,9^

We applied this method in 2001 to forecast breakthrough innovations in 3 clinical areas: Alzheimer disease (AD), cardiovascular disease (CVD), and cancer and the biology of aging. We assessed the accuracy of these forecasts for AD and CVD (we did not have enough resources to assess the third clinical area) in 2017-2018. The present study was approved by the University of Southern California Institutional Review Board and the RAND Corporation Human Subject Protection Committee.

The AD, CVD, and cancer and the biology of aging clinical areas were chosen in 2001 by a multidisciplinary group of geriatric experts as being those most likely to be of the greatest importance to the health of older adults.^7^ Within these clinical areas, potential innovations were identified by horizon-scan literature searches and querying experts. In AD, innovations were proposed and rated in the following categories: improved identification of people at increased risk; primary prevention using compounds based on the amyloid hypothesis; primary prevention using existing or new drugs; treatment of established disease by vaccine, secretase inhibitors, antioxidants, anti-inflammatories, or selective estrogen receptor modifiers; and treatment of established disease by cognition enhancers. For CVD, innovations were proposed and rated in the following categories: improved disease prevention, noninvasive diagnostic imaging to improve risk stratification, noninvasive imaging to replace coronary angiography, the use of implantable cardiodefibrillators, the use of left-ventricular devices as destination therapy, xenotransplants, therapeutic angiogenesis, transmyocardial revascularization, the use of pacemakers and/or defibrillators to control atrial fibrillation, and catheter-based ablation techniques to control atrial fibrillation. Advances in stroke were not considered as part of the CVD panel.

This pilot study combines an untested, unvalidated combination of a consensus process and group judgment process. As 17 of the 20 years had elapsed since the predictions were made, we decided that this was a reasonable time frame to judge their validity. For each clinical area, we first assembled a new diverse group of experts, intentionally including some experts who served on the panel 16 to 17 years ago, along with new experts. We used a 2-step process to have them assess the accuracy of what was predicted in 2001 to happen in the next 20 years (ie, by 2021) compared with what has happened as of 2017 or 2018. We gave the panelists the following criteria, which we developed, to assess predictions.

1. *Close to correct:* original predictions of breakthroughs that were expected to probably happen by 2021 and have happened or still seem to be on track to likely happen by then; or breakthroughs that had a low probability of occurring by 2021 and still seem unlikely to happen by then but are still active areas of research (ie, the “breakthrough” has not yet proved to be a blind alley and abandoned). Breakthroughs that were judged to be about 50/50 to occur by 2021 are going to need your judgment about whether that 50/50 guess was a good one, based on the status of the breakthrough in terms of continued development.
2. *Directionally correct:* original predictions of breakthroughs that do not meet the criteria of “close to correct” but were not altogether wrong, either. For example, the innovation did happen, but it does not look like it will have as big an effect as originally thought (eg, not a “dramatic improvement”). Or the breakthrough has had more-than-anticipated difficulties in overcoming basic biological or implementation challenges but remains very much an area of active research.
3. *Not correct:* original predictions that were simply off—the innovation did not pan out as hoped or was a blind alley, and it is not a subject of much active research anymore.

The experts first rated the predictions at home and without group discussion using an internet survey platform, and then they performed a second rating at the face-to-face panel meeting after group discussion. We also explicitly assessed the possibility of false-negatives of the 2001 predictions, that is, innovations that occurred but were not predicted by the 2001 process. For the AD panel, there was unanimity regarding the missed innovation and no vote was needed. For CVD, 11 potential innovations were proposed by the individual panelists. These innovations were discussed at the face-to-face meeting, and then we conducted a 1-round anonymous voting process and required a two-thirds majority as necessary to characterize something as a missed innovation.

We then had the experts give a global judgment, considering accuracy of predictions and missed innovations, on the 2001 predictions using the familiar A-B-C-D-F grading marks used in school.

## Results

In 2001, the AD expert panel was not very optimistic about breakthrough innovations in the next 20 years. No innovation was judged as being even 50% likely of being in routine clinical practice by 2021. The 3 most likely innovations, each judged to be approximately 40% likely to occur, were primary prevention of AD either through treatments based on the amyloid hypothesis or by the use of new or existing drugs working via some other pathway (eg, anti-inflammatory medications, antioxidants, or selective estrogen receptor modulators) and treatment of established AD with cognition enhancers. The first 2 of these predictions were judged to be close to correct in that they were correctly predicted to be unlikely to happen by 2021, but both are still the subject of intense research activity, and there is still optimism that they may be achieved in the intermediate future. The third of these predictions was judged to be directionally correct in that it was correctly predicted to be unlikely to happen by 2021. There are numerous cognition enhancers under development, but the size of the benefit, if in fact any benefit ever is demonstrated, is unlikely to be as large as the 2001 panel predicted. The 2 other innovations assessed in 2001, each judged as being approximately 30% likely to be in widespread use by 2021, were better identification of AD risk via genetic or metabolic analysis and treatment of established AD via any number of agents, such as vaccines and secretase inhibitors. Both of these predictions were judged to be close to correct, as neither of them is likely to happen by 2021, but research continues on both with the expectation that they will ultimately be successful.

There were no predictions judged as being not correct—innovations that did not develop as expected—and not the subject of continued research. The innovation that the 2001 panel missed was improvements in diagnostic imaging, in particular, amyloid β plaque imaging and tau tangle positron emission tomographic imaging. Overall, this process judged 4 predictions as being close to correct, 1 prediction as being directionally correct, no predictions as being incorrect, and 1 innovation being missed (Table 1). The experts considered the overall accuracy of the 2001 forecast to merit a B+ grade. The results of the assessment of accuracy of the 2001 predictions for each potential innovation are presented in Table 2.

**Table 1.** Accuracy of Predictions for Innovations in 20 Years

Category of Accuracy	Disease Predictions, No.	
	Alzheimer	Cardiovascular
Close to correct	4	6
Directionally correct	1	4
Not correct	0	2
Missed innovations	1	2

**Table 2.** Accuracy of Predictions Made in 2001 About Innovations in Alzheimer Disease by 2021^a^

Innovation, Effect, and Cost	Prediction of Likelihood of Occurrence at 20 Years From 2001, %	Results From Expert Panel Assessment of Accuracy
AD better identification of risk		
By genetic profiling and/or metabolic analysis	30	Close to correct
Estimated effect: no direct effect on mortality or morbidity, but it will identify people at higher risk for guided treatment		
AD primary prevention		
By things related to the amyloid hypothesis, eg, vaccine or secretase inhibitor	40	Close to correct
Estimated effect: delay of onset by median 5 y (range, 3-10 y), slow progression by a mild to moderate amount		
AD primary prevention		
By existing or new drugs/compounds, eg, antioxidants, anti-inflammatory agents, or SERMs	40	Close to correct
Estimated effect: delay of onset by 2-5 y, minor effect on progression		
AD treatment of established disease		
By agents such as vaccines, secretase inhibitors, antioxidants, or SERMs	30	Close to correct
Estimated effect: decrease in rate of progression that is mild to moderate		
AD treatment of established disease		
By cognition enhancers	40	Directionally correct, somewhat overoptimistic
Estimated effect: shifts back in time by 6 mo to 2 y but does not modify the disease		

Abbreviations: AD, Alzheimer disease; SERMs, select estrogen receptor modulators.

^a^ Current rate of progress from diagnosis to death is approximately 10 years. Mild slowing of progression is defined as 20% to 25%; moderate, 50%.

In 2001, the CVD expert panel was more optimistic about breakthrough innovations occurring by 2021. Five innovations were considered at least 50% likely to be in widespread clinical use: innovations in noninvasive diagnostic imaging to better identify high-risk patients with either subclinical disease or clinical disease, magnetic resonance angiography as a replacement for coronary catheterization, widespread use of left-ventricular assist devices as destination therapy, and the use of pacemakers and/or defibrillators to control atrial fibrillation. Of these 5 predictions, 2 were judged as being close to correct (noninvasive diagnostic imaging to better risk stratify patients with clinical disease, expanded use of left-ventricular assist devices), 1 prediction was judged as being directionally correct (noninvasive diagnostic imaging to better risk stratify patients with subclinical disease), and 2 predictions were judged as being not correct (pacemakers and/or defibrillators for patients with atrial fibrillation, magnetic resonance angiography as a replacement for coronary angiography). With regard to the latter development, the expert panel noted that a prediction that there would be an innovation in noninvasive imaging to replace coronary catheterization is directionally correct—such imaging is already used in clinical practice in selected tertiary centers. What was not correct in 2001 was the identification of magnetic resonance as the imaging modality that would be successful in this regard; the imaging that is being used in place of coronary angiography is single-photon emission computed tomography.

Of 7 additional forecasts considered to be less likely to be achieved by 2021, 4 were judged to be close to correct and 3 were judged as being directionally correct. Two potential treatments for which there was clinical enthusiasm in 2001—transmyocardial revascularization and therapeutic angiogenesis as a replacement for revascularization (hailed in the title of one 2000 article as “a ‘breakthrough technology’ in cardiovascular medicine”^10^)—were predicted to be very unlikely to be widely used in 2021, and both of these predictions of failure were judged to be close to correct. Overall, 6 of the cardiovascular predictions were judged as being close to correct, 4 were judged to be directionally correct, and 2 were judged to be not correct (Table 1). Two innovations that occurred were not predicted in 2001: the transcatheter approach to valve replacement, particularly the aortic valve (transcatheter aortic valve replacement), and cardiac resynchronization therapy. Of the additional 9 interventions that were considered, the 3 that came closest to reaching a two-thirds majority vote were novel oral anticoagulants, drug-eluting stents, and percutaneous mechanical supports. Statins and primary percutaneous coronary intervention were already in clinical use at the time of the 2001 panel and therefore were not eligible for consideration as breakthrough innovations. The experts considered the overall accuracy of the forecast to merit a B grade. The results of the assessment of accuracy of the 2001 predictions for each potential innovation are presented in Table 3.

**Table 3.** Accuracy of Predictions Made in 2001 About Innovations in Cardiovascular Disease by 2021

Innovation, Effect, and Cost	Prediction of Likelihood of Occurrence at 20 Years From 2001, %	Results From Expert Panel Assessment of Accuracy
Improved disease prevention	40	Close to correct
Estimated effect: 90% reduction in cardiovascular disease	40	Close to correct
Noninvasive diagnostic imaging to improve risk stratification		
General population aged >45 y	15	Directionally correct
Subclinical disease	75	Close to correct
Clinical disease	50	Close to correct
Estimated effect: better identification of high-risk patients leading to effective risk-reduction strategies		
Magnetic resonance angiography (as a replacement for coronary catheterization)	100	Not correct
Estimated effect: replacement for conventional coronary angiography, likely to increase the number of people undergoing the procedure		This concept that noninvasive imaging will replace coronary catheterization for the same clinical indications was judged as being directionally correct; what was incorrect in the 2001 prediction was the belief that magnetic resonance imaging would be the modality
ICDs for clinical disease	30-40	Directionally correct
Estimated effect: life expectancy for people with heart failure gets shifted 6-10 mo; 20% now die of some other cause		
Left-ventricular assist devices	50	Close to correct
Estimated effect: general increase in functioning for people with functional limitations, 50% decrease in heart failure–related hospitalizations, and 20% of patients will have improved 1-y mortality		
Xenotransplants	1-3	Directionally correct
Estimated effect: possibly similar to the benefit from human heart transplants, but several experts thought that the effect would be lower as the population affected is likely to be different		
Therapeutic angiogenesis		
Clinical disease: augmentation for revascularization	Currently used	
Clinical disease: replacement for revascularization	10	Close to correct
Estimated effect: little effect on mortality, decreased number of revascularization procedures by 20%-30%		
Transmyocardial revascularization	0-5	Close to correct
Estimated effect: little effect on mortality, decreased number of revascularization procedures by 20%-30%		
Pacemakers/defibrillators to control atrial fibrillation	50	Not correct
Catheter-based ablation techniques to control atrial fibrillation	20	Directionally correct
Estimated effect: decreased stroke by 50% of the attributable fraction due to atrial fibrillation		

Abbreviations: ICD, implantable cardioverter defibrillators; LVADs, left ventricular assist devices.

In summary, Table 1 shows that across transcatheter aortic valve replacement and cardiac resynchronization therapy, 15 of 17 innovations forecasted were judged to be close to correct or directionally correct. Two were judged to be incorrect, and there were 3 missed innovations.

## Discussion

This pilot study assessed the accuracy of 20-year forecasts about the potential for future innovations in 2 diseases. Now, nearly 20 years later, 5 of 5 predictions about innovations in AD were judged to be close to correct or directionally correct, and none were judged to be incorrect. For CVD, 10 of 12 predictions were judged to be close to correct or directionally correct, while 2 were judged to be incorrect, albeit for one of the innovations, the underlying principle was judged to be directionally correct, with only the specific imaging modality that was designated in the prediction being proved to be incorrect. There was 1 missed innovation in AD and 2 missed innovations in CVD. Thus, across both disease areas, 15 of 17 predictions were judged to be close to correct or directionally correct, 2 were judged to be incorrect, and there were 3 missed innovations.

The results show that these forecasts were good but not perfect. How the accuracy of these results compares with other methods of forecasting future innovations is difficult to assess because, to our knowledge, no other studies have attempted to systematically assess the accuracy of predictions made using a defined method. A search of published studies from 1999 to 2001 about potential future directions in these 2 areas shows that nearly all such studies about future directions either did not make predictions or made only vague predictions. For example, 3 articles published in 2000-2001 about pharmacogenomics and Alzheimer disease,^11^ neuroprotection,^12^ and new frontiers^13^ imply, respectively, that genomic screening will eventually become mandatory for the early diagnosis of Alzheimer disease, antioxidants and antiamyloid treatments are very promising, and gene profiling will eventually engender early prediction of Alzheimer disease. The few publications that were more firm in their predictions tended to be more overly optimistic than the results of our 2001 panel. One 2001 publication predicted that “therapeutics lies just over the horizon,”^14^ and a 2000 article states that various drugs experimental at that time will be used clinically in the near future.^15^ A popular media article quoted a noted Alzheimer researcher in 2000 as “believing that medical practitioners will shortly have on hand not one but several drugs capable of slowing—and perhaps even halting—the progression of the disease.”^16^ Only 1 futures study identified from 2001 about selective estrogen receptor modulators was not optimistic.^17^ The results of a search for cardiovascular medicine breakthroughs revealed much the same result: most studies had no or only vague predictions, even those with “the future” or “predicting the future” in the title.^18,19^ One notable exception was predictions made in “Technological Advances and the Next 50 Years of Cardiology.”^20^ These authors predicted that, by the year 2009, replacement hearts, either battery-powered electromechanical devices or pig hearts, would be commonplace and that by 2024 heart exchange would be the most common type of open-chest surgery. Advances in genomics would allow both precise prediction and a “flood of powerful new pharmaceuticals,”^20^ such as angiogenesis drugs, myogenesis drugs, and vaccines that raise high-density lipoprotein cholesterol levels. Compared with other future predictions of innovations in AD and cardiovascular medicine published at the same time, the forecasts made by our 2001 expert panels seem quantitatively more accurate. How the accuracy of this method compares with newer methods of forecasting, such as big data or prediction markets, is unknown because, to our knowledge, accuracy studies for newer methods have not yet been published. Our results provide a benchmark to compare the results of such studies when they become available.

In terms of the missed innovations, we speculate that this omission may have owed to the 2001 panels not containing the proper expertise. So, the 2001 AD panel did not contain an expert in imaging and the 2001 CVD panel did not contain an interventional cardiologist. It is possible that this lack of representation contributed to having missed predicting innovations in imaging for AD and transcatheter aortic valve replacement, respectively. No future innovation article published in 1999-2001 identified in our literature search predicted new imaging techniques for AD or transcatheter aortic valve replacement.

### Limitations

This study has a number of limitations, among them being that we were only able to do the assessment of accuracy for 2 clinical disease areas, that there are no data on other methods with which to compare these results, and the known variability in expert panel processes, with the latter limitation meaning that a different group of experts would have likely come up with at least some differences in predicted innovations and/or differences in the assessments of the accuracy of those predictions. Even among the experts who participated in our panels there was individual disagreement about many topics, such as whether oral anticoagulants have produced a substantial enough result to be considered a breakthrough and whether the low predicted likelihood for xenotransplants was directionally correct (xenografts were predicted in 2001 with a very low likelihood and have not happened yet, but research continues, and they might yet happen someday) or incorrect (xenografts will never happen). Changes in panel composition could result in different summary results. Determining how to select experts for future forecasting panels is a subject needing additional study, similar to studies of the effect of panel composition on group judgments of appropriateness.^21^ In contrast to studies of appropriateness, the role of nonscientists needs investigation for future innovation predictions, such as experts in philanthropy, health care administration, or the financial markets. Nevertheless, our results are strengthened by having a defined method to select the panelists and generate the forecasts, and we were able to assess the accuracy for 2 different clinical conditions and achieve similar results.

## Conclusions

We report herein the results of a systematic assessment of forecasts of future innovations in AD and CVD. The accuracy of these forecasts was judged as successful by experts but missed some key innovations. These predictions seemed to be more accurate than other published predictions from the same period.

Why does the ability to try and make future forecasts matter? Over the past 50 years, life expectancy has increased greatly, along with significant enhancements in the functional capacity of older persons. These gains have been driven by advances in public health and medical treatment. But the longer lives people now enjoy come with social and fiscal consequences. More people are qualifying for old-age entitlement programs, and they remain in these programs longer than people did in the past. Medicare spending alone is projected to almost double as a share of national income from 3.7% today to 7.3% in 2050. An understanding about how medical technology will exacerbate—or alleviate—these trends is critical to making future progress. Based on these results, one federal agency (the National Institute on Aging) has sponsored new expert panel forecasts of innovations in several disease areas. Features of this method that we speculate may contribute to improved accuracy of forecasts include having multiple experts with a diversity of experience, a horizon-scanning literature search, a face-to-face panel meeting, and multiple rounds of quantitative assessments, with feedback of group results between rounds.
